# Detection of EBV DNA in clinically ill patients from 2013 to 2023 in Beijing, China

**DOI:** 10.3389/fmicb.2025.1651320

**Published:** 2025-11-03

**Authors:** Ze Su, Ziran Wang, Jie Yi, Yu Chen, Han Zhang, Dong Zhang, Lingjun Kong, Juan Du, Qiwen Yang, Rui Zhang, Yali Liu

**Affiliations:** ^1^Department of Clinical Laboratory, Peking Union Medical College Hospital, Chinese Academy of Medical Sciences & Peking Union Medical College, Beijing, China; ^2^Department of Clinical Laboratory, Zibo First Hospital, Zibo, Shandong, China

**Keywords:** Epstein–Barr virus DNA, positive rate, EBV-associated diseases, infectious mononucleosis, real-time PCR

## Abstract

**Objective:**

To investigate the epidemiological characteristics and infection patterns of Epstein–Barr virus (EBV) among patients at Peking Union Medical College Hospital (PUMCH) between 2013 and 2023, with a concurrent analysis of the clinical manifestations and laboratory parameters observed in patients diagnosed with EBV-associated infectious mononucleosis (IM).

**Methods:**

A retrospective analysis was conducted on data from 76,135 patients who underwent EBV DNA testing at Peking Union Medical College Hospital between 2013 and 2023. Additionally, clinical data from 152 patients diagnosed with IM from 2013 to 2024 were collected, with their clinical manifestations and laboratory parameters thoroughly evaluated.

**Results:**

The overall EBV DNA positivity rate was 6.77%, with significant variations observed across different years and age groups. The detection rate of EBV DNA in males was significantly higher than in females, with the highest detection rate observed in individuals aged over 71 years. A decline in EBV DNA detection frequency was noted during the post-COVID-19 pandemic period (2020–2023). Among EBV-related clinical entities, the highest EBV DNA positive rate (37.4%) was observed in patients with IM. In this cohort, liver function abnormalities were common: elevations of alanine aminotransferase (ALT) and aspartate aminotransferase (AST) by 1- to 5-fold above the upper limit of normal were present in 61 (57%) and 41 (59%) cases, respectively. Immunophenotypic analysis revealed a marked increase in the CD8 + DR+/CD8 + ratio in 67 patients (99%), accompanied by a reduction in CD19 + B-lymphocyte percentage in 62 cases (90%), indicating pronounced immune activation and B-cell suppression characteristic of acute EBV infection.

**Conclusion:**

The positive rates of EBV DNA showed sex differences and significant fluctuations between 2013 and 2023, without displaying a consistent upward trend. Persistent surveillance and heightened clinical awareness (laboratory parameters, such as ALT, AST and CD8 + DR+/CD8+) are warranted, particularly regarding IM attributable to EBV.

## 1 Introduction

Epstein–Barr virus (EBV), a member of the *Gammaherpesvirinae* subfamily, is an enveloped virus characterized by an icosahedral capsid comprising 162 capsomeres. The mature virus particle contains one linear double-stranded DNA genome. Its primary cellular tropism includes B lymphocytes and epithelial cells. As the first human virus definitively linked to oncogenesis, EBV has achieved a global seroprevalence exceeding 90%. The first exposure to EBV is usually in childhood. Primary infection typically occurs during childhood and is often asymptomatic. Following initial infection, EBV establishes lifelong latency predominantly within memory B cells, maintaining a persistent, largely asymptomatic reservoir that evades immune clearance and remains incurable (Nowalk and Green, 2016). In addition to its well-established associations with infectious mononucleosis (IM), nasopharyngeal carcinoma (NPC), and Burkitt lymphoma, EBV is increasingly associated with lymphoproliferative disorders (EBV-LPD), chronic active EBV infection (CAEBV), and a growing number of other immunological and oncologically significant conditions (Young and Rickinson, 2004; Wang et al., 2016).

At present, the main detection methods of EBV include EBV-related antibody test, heterotypic lymphocyte typing, EBV nucleic acid quantification, and Epstein–Barr virus-encoded RNA (EBER) *in situ* hybridization (Engelmann et al., 2018; Farrell, 2019). In this study, real-time quantitative PCR was employed to quantify EBV DNA load, a method offering high precision in viral load assessment. This approach provides clinically relevant data that are instrumental for accurate diagnosis and for guiding stage-specific therapeutic interventions. To investigate the EBV DNA positivity rate and the immune impairment in IM, and to provide insights for clinical management, the EBV DNA testing results and the clinical data of 152 patients with IM between 2013 and 2023 were retrospectively analyzed.

## 2 Materials and methods

### 2.1 Study population

From January 1st, 2013 to December 31st, 2023, a total of 76,135 patients who underwent EBV DNA testing at Peking Union Medical College Hospital (PUMCH) were included in this study following the exclusion of duplicate cases. The study cohort comprised both outpatient and inpatient individuals referred from various clinical departments, including Oncology, Hematology, Infectious Diseases, Respiratory Medicine, Rheumatology and Immunology, Pediatrics, and other specialties, thereby ensuring a broad representation of clinical presentations across the institution during the 10-year study period. To exclude the repeated samples, serial test results from the same patient were evaluated using predefined criteria: only the first test result was retained as the baseline. Subsequent results were considered indicative of a new infection only if discrepant from the initial result. For patients with an initial positive result (≥400 copies/mL), any subsequent negative result (<400 copies/mL) was not counted as a new event, and only the initial positive result was recorded. Repeat positive results were excluded; only the first positive test was included. In cases of multiple negative results, a single negative was retained. Applying the these criteria, 36,849 EBV DNA test results from male patients aged 2 days to 104 years and 39, 286 from female patients aged 6 days to 99 years were included in the final analysis.

According to the Expert Consensus on the Diagnosis and Treatment Principles of diseases associated with EBV infection in children, the diagnosis of IM requires the presence of any three of the following clinical manifestations in conjunction with at least one non-specific laboratory criterion. Clinical manifestations includes: (1) Fever; (2) Pharyngitis; (3) Enlarged cervical lymph nodes; (4) Hepatomegaly; (5) Splenomegaly; (6) Eyelid edema. Non-specific laboratory tests: (1) Percentage of atypical lymphocytes in peripheral blood≥0.10; (2) Percentage of atypical lymphocytes in peripheral blood in children aged 6 years or older ≥0.10; Cases of co-infection or cases with incomplete case information were excluded. A final cohort of 152 patients meeting the diagnostic criteria for IM was included in the study.

### 2.2 Ethics statement

The study adhered to the ethical principles of the Declaration of Helsinki and was approved by the Ethics Committee of Peking Union Medical College Hospital, Chinese Academy of Medical Sciences (No. I-23PJ1939).

### 2.3 Sample collection

A 2 mL whole blood sample was collected in an EDTA-K₂ anticoagulant tube and centrifuged at 3,000 rpm for 5 min to isolate plasma, which was subsequently transferred into an EP tube for further processing. Then the EP tubes were placed in the refrigerator at 4 °C. The plasma portion of the sample was used for real-time PCR detection. In 1–2 mL of venous blood sample from a standard tube, serum was routinely separated, stored at 2–8 °C, with the serum portion used for EBV antibody testing.

### 2.4 EBV DNA testing

DNA extraction, PCR amplification and detection, and results interpretation were performed in accordance with the manufacturer’s instructions. EBV DNA detection reagent (Sun Yat-sen University DAAN Gene Co, Ltd.) was used for nucleic acid extraction and quantitative detection. The Roche Light Cycler 480 II real-time PCR instrument was used for the detection of EBV DNA concentration. The plasma portion of the sample was used for real-time PCR detection. An EBV DNA level exceeding 400 copies/mL was defined as a positive test result. The positivity rate reported in this study reflects the proportion of samples testing positive for EBV DNA.

### 2.5 EBV antibody testing

EBV antibody detection was performed strictly in accordance with the manufacturer’s instructions. Enzyme-linked immunosorbent assay (ELISA) was used for the determination of EBV antibodies, and the manual method was performed using the SUNRISE enzyme labeling instrument of TECAN (Switzerland) and the MultiWash microplate washer of MOLECULAR DEVICES (United States). The instrumental method was performed using the Addcare Elisa 600, a fully automated enzyme immunoassay workstation, for the determination of EBV antibodies.

### 2.6 Lymphocyte subpopulation testing

Lymphocyte Subpopulation was analyzed using the BD FACS CANTO II Flow Cytometer.

### 2.7 Testing of other indicators

The BECKMAN AU series automatic biochemical analyzer was used for the determination of ALT, AST, GGT, and LDH, and blood routine indexes were detected by Siemens ADVIA 2120 blood cell analyzer from Germany and SYSMEX XE-5000 hematology analyzer from Japan.

### 2.8 Statistical analyses

The data collected in PUMCH’s Laboratory Information System (LIS) were exported and managed through Excel 2019 software (Microsoft, United States). Statistical analyses were performed using SPSS (version 24.0; SPSS, Chicago, IL, United States). Graphs were created using GraphPad Prism software (version 9.0). In this study, differences between groups were compared by the chi-squared (χ^2^) test. For all statistical analyses, *p* < 0.05 was considered statistically significant.

## 3 Results

### 3.1 EBV DNA positive rates

**Figure 1:**
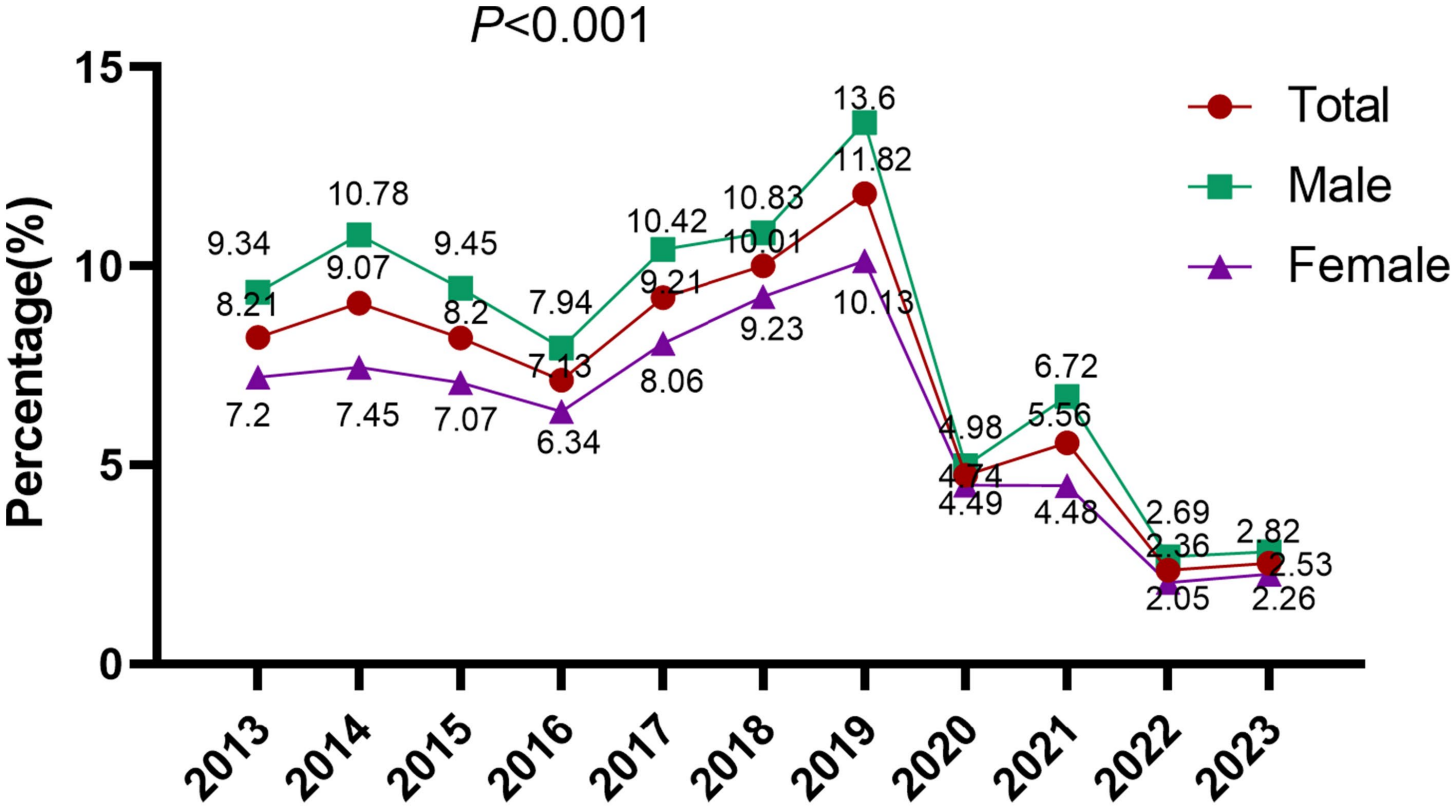
The positive rates of EBV DNA testing in a total of 76,135 samples from 2013 to 2023.

The overall EBV DNA positive rate across the study period was 6.77% (5,154/76,135). Annual stratification revealed marked temporal variation, with positive rates fluctuating between 2.36 and 11.82%: 8.21% (284/3,458) in 2013, 9.07% (426/4,695) in 2014, 8.20% (501/6,109) in 2015, 7.13% (447/6,272) in 2016, 9.21% (651/7,067) in 2017, 10.01% (759/7,579) in 2018, 11.82% (920/7,785) in 2019, followed by a marked decline to 4.74% (238/5,025) in 2020, 5.56% (424/7,627) in 2021, 2.36% (209/8,866) in 2022, and 2.53% (295/11,652) in 2023. The difference between the groups was statistically significant (*p* < 0.001). The period from 2017 to 2019 was defined as the pre-COVID-19 era, while 2020 to 2022 was designated as the COVID-19 pandemic period for comparative analysis (Wang et al., 2023). The EBV DNA positivity rate during the COVID-19 pandemic period (2020–2022) was markedly reduced at 4.05% (871/21,518), compared to 10.39% (2,330/22,431) in the pre-COVID-19 period (2017–2019), with the difference reaching high statistical significance (*p* < 0.001), indicating a substantial decline in EBV detection following the onset of the pandemic (Figure 1).

### 3.2 Positive rates of EBV DNA across age groups and genders

**Figure 2:**
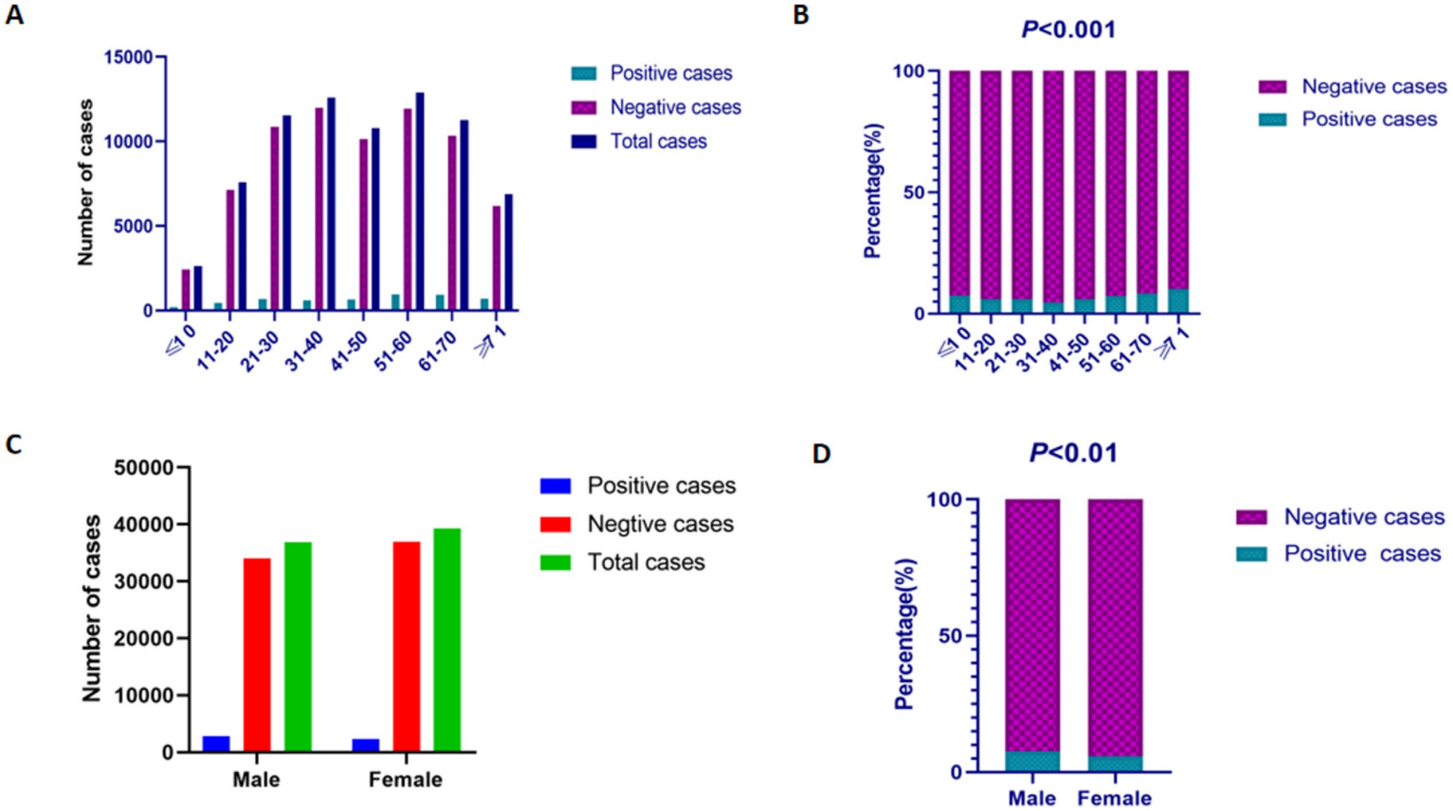
The prevalence of EBV DNA in different gender and age groups. **(A)** The results of EBV DNA testing in different age groups. **(B)** The positive rates of EBV DNA testing in different age groups. **(C)** The results of EBV DNA testing in male and female patients. **(D)** The positive rates of EBV DNA testing in male and female patients.

The positive rates of EBV DNA varied across age groups: 7.48% (196/2,621) in children aged 0–10 years, 6.01% (456/7,590) in individuals aged 11–20 years, 5.94% (685/11,540) in those aged 21–30 years, 4.72% (594/12,589) in the 31–40 years group, 5.97% (643/10,775) in the 41–50 years group, 7.36% (948/12,877) in the 51–60 years group, 8.25% (930/11,267) in the 61–70 years group, and 10.21% (702/6,876) in individuals aged ≥71 years. The EBV DNA positive rates differed significantly across age groups (*p* < 0.001), exhibiting a non-linear distribution with age. The highest positive rate was observed in individuals aged 70–104 years (10.21%), whereas the lowest rate was recorded in those aged 31–40 years (4.72%) (Figures 2A,B). Among the 76,135 samples, the positive rate of EBV DNA in males (7.69%, 2,834/36,849) was significantly higher than that in females (5.91%, 2,320/39,286) (*p* < 0.01) (Figures 2C,D).

### 3.3 Positive rates of EBV DNA across different clinical diagnoses

Patients who presented to our hospital between January 1, 2013, and December 31, 2023, were categorized according to their clinical diagnoses, and the prevalence of EBV DNA positive was comparatively analyzed across disease groups. The top ten EBV-associated conditions are ranked in descending order of EBV DNA positive rates. IM exhibited the highest positive rate at 37.4%, followed by NPC (26.6%) and nasopharyngeal mass (16.3%) (Table 1).

**Table 1:** The top ten EBV-associated conditions with EBV DNA detection rates (*n* = 76,135).

Diseases	Positive cases/total cases	Positive rates
Infectious mononucleosis	64/171	37.43%
Nasopharynx cancer	183/687	26.64%
Nasopharyngeal mass	57/350	16.29%
Viral infection	258/1,647	15.66%
Lymphoma	310/2,555	12.13%
Pulmonary infection	467/3,915	11.93%
Lymphadenectasis	179/2,073	8.63%
Fever	1,423/18,888	7.53%
SLE	195/3,263	5.98%
Ulcerative colitis	54/1,342	4.02%

## 4 A total of 152 patients clinically diagnosed with IM between 2013 and 2024 were included in the analysis

### 4.1 Clinical symptoms of IM patients

**Figure 3:**
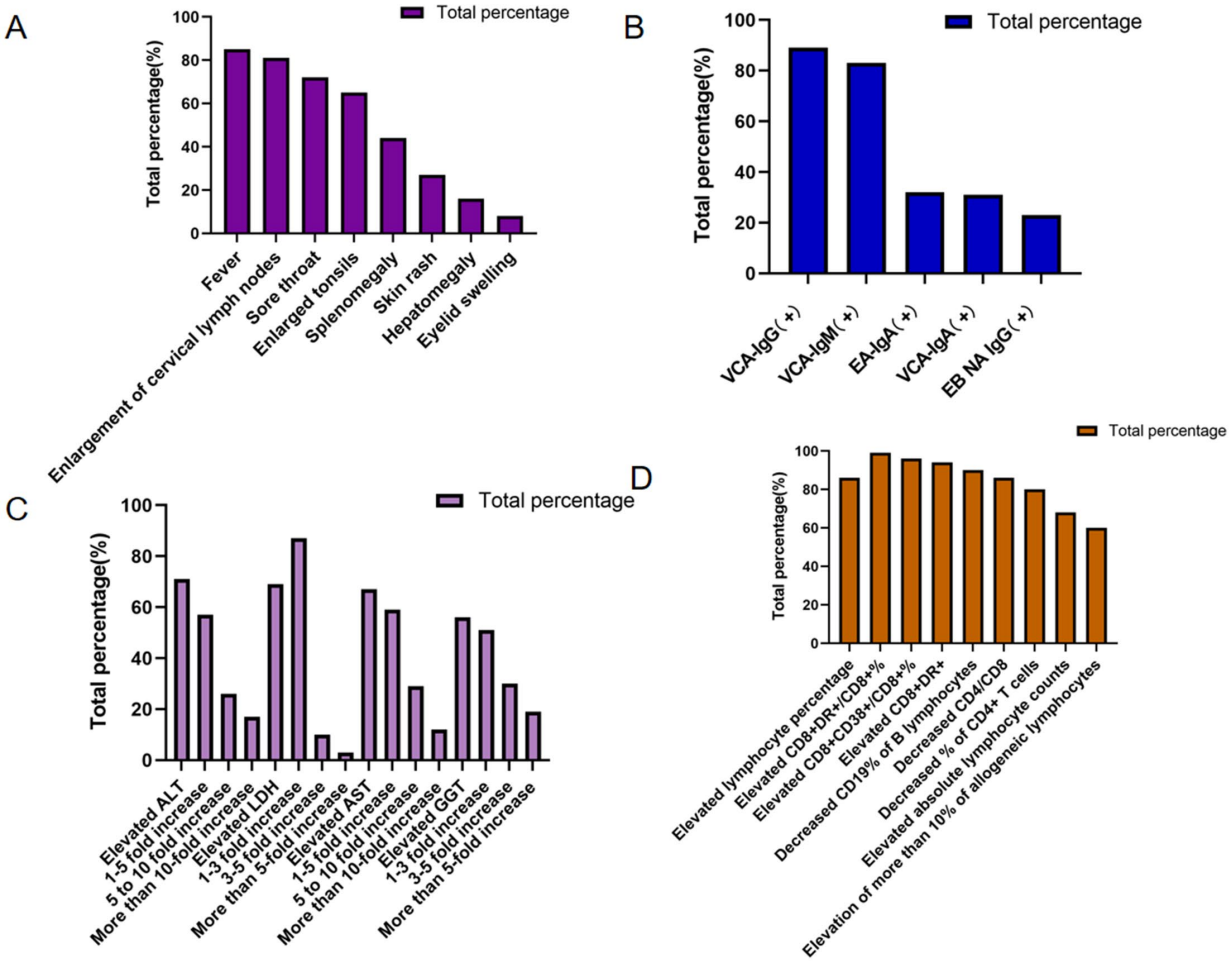
Percentage of clinical data for IM patients. **(A)** Clinical symptoms of IM patients. **(B)** EBV antibody among IM patients. **(C)** Liver function of IM patients. **(D)** Lymphocyte features of IM patients.

In Clinical manifestations in the cohort of 152 patients with IM revealed that fever, cervical lymphadenopathy, and pharyngitis were the predominant symptoms, present in 85, 81, and 72% of cases, respectively. Fever was most frequently observed with a duration of 1–2 weeks (34 cases, 45%) and a peak temperature exceeding 38 °C (70 cases, 95%). Other common clinical features included tonsillar enlargement, hepatosplenomegaly, cutaneous rash, and periorbital edema. Notably, exudative tonsillitis, characterized by pseudomembranous or purulent secretions on the tonsillar surface, was present in 39 patients (68%) (Figure 3A).

### 4.2 EBV antibody among IM patients

The results showed that among 152 patients with IM, the positive rates of EBV antibodies were EBV capsid antigen IgG (VCA-IgG) (89%), EBV capsid antigen IgM (VCA-IgM) (83%), EBV early antigen IgA (EA-IgA), EBV capsid antigen IgA (VCA-IgA) (31%), and anti-EBV nuclear antigen IgG (EBNA IgG) (23%). The highest positive rate was EBV VCA-lgG (89%), which is consistent with acute or past EBV infection, supporting the etiological link between EBV and IM. In contrast, the antibody against EBNA IgG exhibited the lowest positive rate, detected in only 23% of the patients (Figure 3B).

### 4.3 Liver function of IM patients

Liver function was assessed using selected biochemical markers, including ALT, LDH, AST, and GGT. Among 152 patients with IM, all four markers demonstrated varying degrees of elevation, the percentages of elevated ALT, LDH, AST, and GGT were 107 (71%), 70 (69%), 69 (67%), and 47 (56%), respectively, indicating hepatic involvement during acute EBV infection. Among those with elevated transaminases, mild-to-moderate increases (1- to 5-fold above the upper limit of normal) in ALT and AST were the most common, accounting for 61 (57%) and 41 (59%) of affected cases, respectively. On the other hand, GGT and LDH were elevated 1- to 3-fold in 24 (51%) and 61 (87%) of the affected cases, respectively, further supporting the presence of hepatocellular involvement and systemic cellular turnover in patients with IM (Figure 3C).

### 4.4 Lymphocyte features of IM patients

Lymphocyte-related parameters were further analyzed in the cohort of 152 patients with IM. Lymphocytosis, defined by an increased lymphocyte percentage, was observed in 131 cases (86%), while elevated absolute lymphocyte counts were present in 100 cases (68%). Additionally, peripheral blood atypical lymphocytosis was documented in 59 patients with available morphological assessment (60%), consistent with the hematological hallmark of acute EBV infection. Regarding lymphocyte subpopulations, the CD8 + DR+/CD8 + % ratio was elevated in 67 cases (99%), the CD8 + CD38+/CD8 + % was increased in 65 cases (96%), and CD8 + DR + expression was heightened in 60 cases (94%). Conversely, B-lymphocyte CD19% was reduced in 62 cases (90%), the CD4/CD8 T-cell ratio was decreased in 60 cases (86%), and CD4 + T-cell percentage was diminished in 56 cases (80%) (Figure 3D).

**Figure 4:**
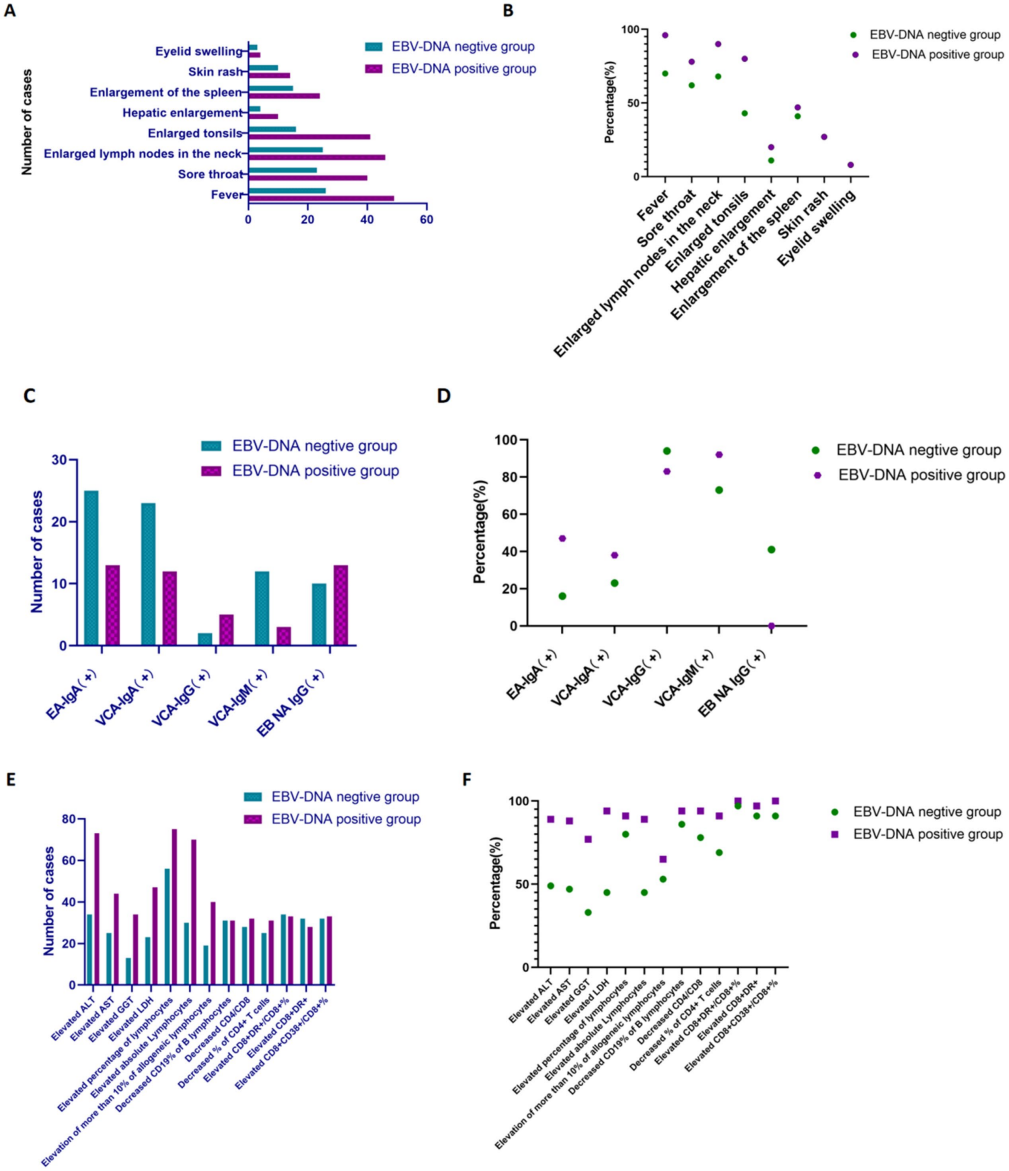
The comparison of various indicators with IM patients between EBV DNA-positive and EBV DNA-negative groups. **(A,B)** Clinical manifestations of IM patients in subgroups. **(C,D)** EBV antibodies among IM patients in subgroups. **(E,F)** Liver function and lymphocyte features of IM patients in subgroups.

**Figure 5:**
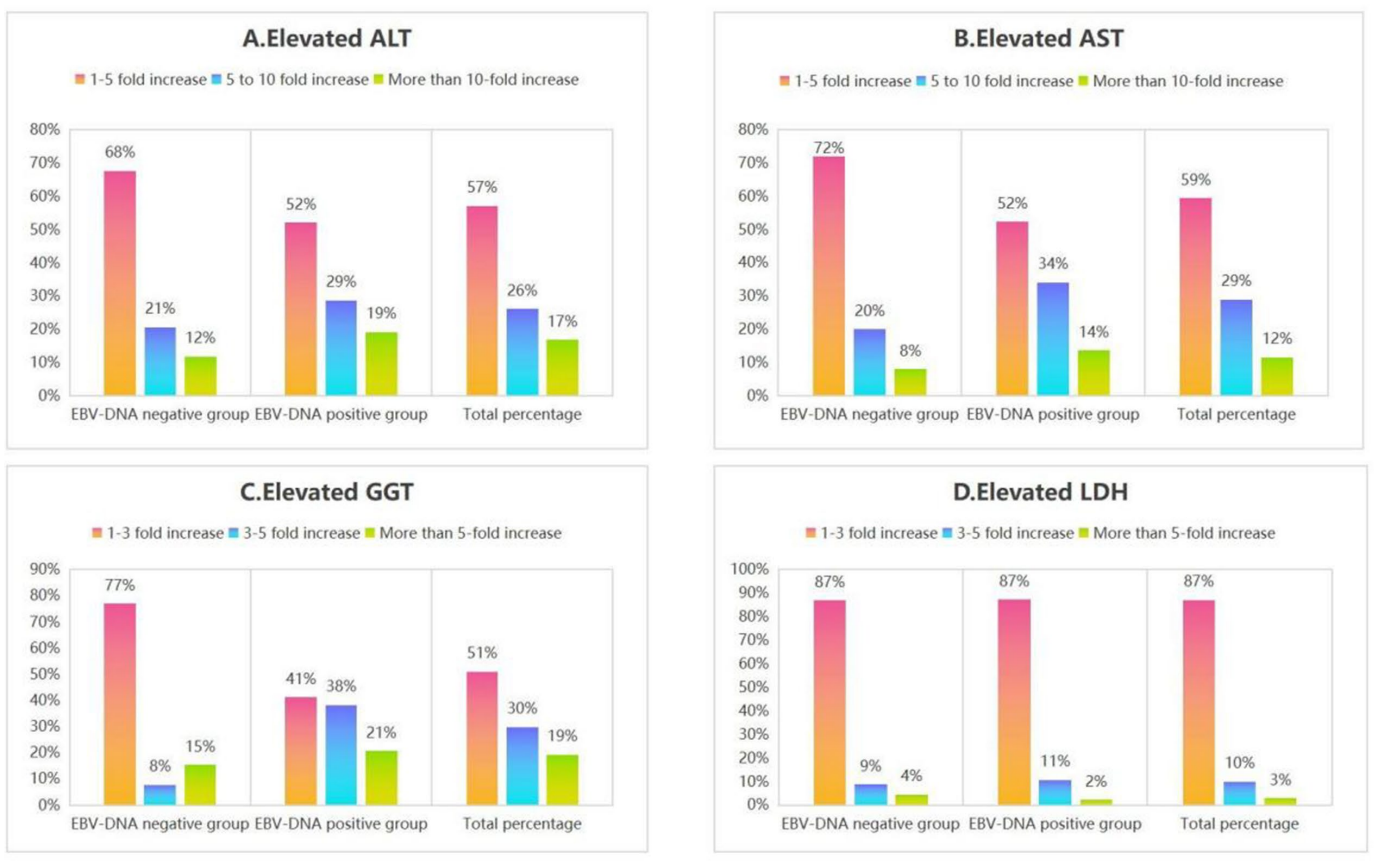
The percentage of elevated liver function indices (ALT, AST, GGT, and LDH) in subgroups of IM patients.

One hundred and fifty-two patients with IM were divided into two subgroups (EBV DNA-positive and EBV DNA-negative groups) based on virological test results. Comparison of the EBV DNA-negative group with the EBV DNA-positive group (Figures 4, 5; Table 2).

**Table 2:** Relationship between EBV antibodies and liver function.

Liver function indicators	EA-IgA		VCA-IgA		VCA-IgG		VCA-IgM		EBNA IgG	
	Positivity	Negativity	Positivity	Negativity	Positivity	Negativity	Positivity	Negativity	Positivity	Negativity
Elevated ALT	90%	55%	90%	54%	70%	71%	74%	53%	29%	74%
Elevated AST	62%	42%	65%	37%	41%	57%	47%	40%	29%	52%
Elevated GGT	48%	29%	55%	20%	27%	43%	32%	33%	29%	39%
Elevated LDH	62%	47%	70%	43%	44%	43%	49%	47%	29%	61%

### 4.5 Clinical symptoms of IM patients in subgroups

Within the context of our research, the frequencies of clinical manifestations, including fever, sore throat, enlarged cervical lymph nodes, enlarged tonsils, hepatosplenomegaly, and enlarged spleen were higher in the EBV DNA-positive group compared with the EBV DNA-negative group among patients with IM (Figures 4A,B). It is worth noting that EBV viral load can not only serve as a biological marker of active infection, but may also reflect the clinical severity of the disease to a certain extent.

### 4.6 EBV antibody among IM patients in subgroups

In the context of our study, the positivity rates of EA-IgA, VCA-IgA, and VCA-IgM in the EBV DNA-positive group were higher than those in the EBV DNA-negative group among the patients with IM (Figures 4C,D). Following infection with EBV, the human body generates specific antibodies directed against various viral antigens. Notably, during primary EBV infection, IgM antibodies targeting the VCA are typically the first to emerge, this observation suggests that patients may have either an active EBV infection or a recently acquired infection. A definitive diagnosis requires additional evaluation in combination with clinical manifestations.

### 4.7 Liver function and lymphocyte features of IM patients in subgroups

Furthermore, regarding laboratory parameters, the proportions of patients with liver function impairment (elevated ALT/LDH/AST/GGT), elevated lymphocyte percentage, and peripheral blood reactive lymphocytes >10% were higher in the EBV DNA-positive group compared to the EBV DNA-negative group. Regarding lymphocyte subpopulations, the EBV DNA-positive group exhibited a higher proportion of abnormal immune activation markers (elevated CD8 + DR+/CD8 + %, elevated CD8 + DR+, elevated CD8 + CD38+/CD8 + %, decreased CD19% of B lymphocytes, decreased CD4/CD8, decreased% of CD4 + T cells) compared to the EBV DNA-negative group. The incidence of immune imbalance was higher in the EBV DNA-positive subgroup, suggesting that immune dysfunction associated with active viral replication is more pronounced in the EBV DNA-positive group, further supporting the correlation between EBV replication intensity and the severity of host immune abnormalities (Figures 4E,F).

### 4.8 Elevated ALT/AST/GGT/LDH features of IM patients in subgroups

Additionally, among the included patients with IM, the 1 to 5-fold elevations in ALT and AST were the most prevalent pattern in both the EBV DNA-positive and EBV DNA-negative groups. Similarly, 1 to 3-fold elevations in GGT and LDH were the predominant findings across both subgroups (Figure 5), as demonstrated in Section 4.3, acute EBV infection may be associated with a certain degree of hepatic dysfunction. This finding suggests that there is likely no significant correlation between EBV-induced hepatic injury and EBV DNA levels.

## 5 Relationship between EBV antibodies and liver function

This study analyzed 152 patients diagnosed with IM. Among patients who tested positive for both EA-IgA and VCA-IgA, the incidence of elevated ALT was highest, with 90% of individuals in this group exhibiting abnormal ALT levels. This suggests that EBV-specific antibody profiles may be associated with liver function parameters, particularly EA-IgA and VCA-IgA (Table 2).

## 6 Discussion

EBV is known to naturally infect only humans, making them the exclusive host for this virus (Fujiwara, 2014; Nowalk and Green, 2016). EBV proliferates in epithelial cells in the pharynx before gaining access to and infecting B lymphocytes. The majority of individuals infected with EBV remain asymptomatic or exhibit nonspecific symptoms, making the infection easily overlooked in clinical practices. However, accumulating evidence has demonstrated a significant association between EBV infection and IM, NPC and other diseases, even in asymptomatic EBV-infected patients (Wang et al., 2016). Consequently, early, highly sensitive, and accurate detection and continuous monitoring of EBV infection have become crucial in clinical practice for timely risk assessment, diagnosis, and management of associated diseases. The EBV nucleic acid load test enables differentiation between low-level viral replication, typically observed in asymptomatic EBV carriers, and high-level replication, which is predominantly associated with active infection and various EBV-related diseases (Farrell, 2019). Real-time fluorescence quantitative PCR is the most widely used method for monitoring EBV nucleic acid load, offering high sensitivity and specificity.

Indeed, EBV infection presents with a wide range of clinical symptoms. According to a review of literature over the past 5 years, EBV infection can manifest as IM, characterized by the “triad” of fever, pharyngitis, and lymphadenopathy; multiple sclerosis, characterized by chronic inflammation of the central nervous system and neurodegenerative disease (Cao et al., 2024); and systemic lupus erythematosus (SLE), characterized by vasculitis and cardiovascular involvement (Chen et al., 2024). Additionally, tumors associated with EBV infection include Burkitt’s lymphoma, Hodgkin’s lymphoma, NPC, gastric cancer, and lymphoepithelial-like lung cancer (Cao et al., 2024).

Since its discovery in 1964, EBV has been increasingly recognized as a pathogen associated with a wide spectrum of malignancies worldwide (AbuSalah et al., 2020). EBV type 1 is the most prevalent strain worldwide. Co-infection with EBV type 1 and type 2 is not uncommon, particularly in regions where EBV type 2 is more prevalent and among immunocompromised populations (Neves et al., 2015; Smith et al., 2019). In studies comparing the prevalence of different EBV strains in EBV-associated cancers, EBV type 1 is identified as the predominant variant (Neves et al., 2017; Montes-Mojarro et al., 2020a; Montes-Mojarro et al., 2020b). However, a case–control study of Portuguese Caucasian patients found that, despite the prevalence of EBV type 1 in the region, EBV type 2 was more strongly associated with NPC risk (Neves et al., 2015). Furthermore, the incidence of specific EBV-associated cancers is strongly associated with geographic regions. Examples include Burkitt lymphoma (BL) in regions with holoendemic *Plasmodium falciparum* malaria in Sub-Saharan Africa, NPC in East Asia, and natural killer (NK)/T-cell lymphoma (NKTCL) in East Asia, Central America, and Western South America. Indeed, geographic distribution is a far stronger determinant of viral genome clustering than the specific disease phenotype (Farrell and White, 2021; Briercheck et al., 2024). Meanwhile, in China, IM, NPC, and nasopharyngeal masses are notably prevalent.

Infection typically occurs during early childhood, with most children in developing countries acquiring EBV and becoming seropositive by the age of five. While onset of infection is delayed in areas with greater socioeconomic development, the vast majority of adults ultimately become infected. Our research found an EBV DNA positive rate of 7.48% in children aged 0–10 years. The prevalence of EBV DNA reached its peak in individuals aged over 71 years, while the lowest detection rate occurred in the 31–40 years age group. This suggests that the level of EBV-DNA may be associated with the immune status and age of individuals, warranting further investigation.

According to this study, the positive rate of EBV DNA in the PUMCH in the past 10 years changed markedly but without a rising trend. Although the year-on-year fluctuations in EBV DNA positivity rates were statistically significant (*p* < 0.001), the overall trend remained relatively stable throughout the 10-year period. The EBV DNA positivity declined after the COVID-19 period, falling from 10.39 to 4.05%. This may be related to the fact that social isolation measures taken since the COVID-19 outbreak, including mandatory wearing of masks in public places, cancelation of social gatherings, intensified restrictions on intra- and inter-city transportation, and home isolation, have successfully prevented the spread of COVID-19, which has also reduced interpersonal contact to a certain degree, thereby inhibiting the spread of EBV in society (Lieberman, 2014). The detection rates for males and females were 7.69 and 5.91%, respectively, and the differences were statistically significant (*p* < 0.01), suggesting a potentially higher susceptibility to EBV infection among males compared to females. Current national literature indicates that the prevalence of EBV infection is higher in males than in females, which is consistent with our findings and suggests a possible gender-based disparity in infection risk or immune response. However, a study by Bethany G. observed higher EBV viral loads in females compared to males (Everett et al., 2014), a discrepancy that may stem from variations in population demographics.

A significant variation in EBV DNA positive rates was observed across age groups (*p* < 0.001), with the highest prevalence detected in individuals aged over 71 years. This trend may be attributed to age-related immune decline, leading to reduced control of latent viral reactivation in the elderly population. This observation underscores the importance of prioritizing EBV testing in elderly patients to aid in diagnosis and management. This is consistent with the findings of Dou et al. (2024), who noted that the highest positive rate of EBV DNA detection was at ≥80 years of age at 10.99%. Meanwhile, the positive rate was also higher in the group of children aged 0–10 years. It has been reported that EBV mainly affects school-age children and adolescents in our country (Liu et al., 2024). Several studies have indicated that the incidence of primary EBV infection is particularly high among preschool children under the age of six. This heightened susceptibility may be attributed to the immaturity of their immune systems and relatively weaker immune function compared to older individuals. In contrast, adults typically benefit from a more developed immune surveillance capacity (Zhang et al., 2020).

Following EBV infection, the host produces antibodies specific to different antigens. The presence of antibodies in serum reflects the complex immune response of the host to the virus (Cao et al., 2024). EBV DNA load measurement is widely used in the diagnosis of EBV-related diseases, disease monitoring, assessment of treatment efficacy, and prognosis evaluation. In the early stages of IM infection, cases with prominent clinical symptoms typically exhibit higher EBV DNA loads in serum is typically elevated (Shi et al., 2021; Li et al., 2022). We employ real-time PCR to detect EBV DNA in plasma and an enzyme-linked immunosorbent assay (ELISA) to detect EBV antibodies in serum. However, the positive rate of EBV DNA detection using plasma samples has consistently been lower than the positive rate reported in studies using EBV serology to detect EBV antibodies (Chen et al., 2016). This lower positive rate may result from both improved control of EBV infection and methodological differences. Plasma EBV DNA testing detects active infection, while serological assays detect persistent antibodies. Studies have shown that EBV DNA testing is superior to serologic testing in the diagnosis of active infections; patients with active EBV infections or EBV-associated malignant tumors usually have high EBV DNA loads in their plasma (Mu et al., 2017). While EBV serological testing offers the advantages of rapid turnaround and relatively low cost, its utility in monitoring disease dynamics is limited. Due to the persistence of antibodies, serology cannot accurately reflect real-time viral load or changes in clinical condition. As a result, its diagnostic value for assessing disease activity or treatment response is inferior to that of quantitative EBV DNA testing, which provides a more direct and dynamic measure of viral replication (Chen, 2018).

Although EBV nucleic acid load testing exhibits limited negative predictive value in the diagnosis of IM, it serves as a valuable adjunct when antibody results are ambiguous. More importantly, it offers a direct measure of viral activity, enabling clinicians to monitor fluctuations in viral load and assess the dynamic course of infection (National Collaborative Group on EB Virus Infection in Children and Editorial Board of Chinese Journal of Experimental and Clinical Virology, 2018). Because of the persistent presence of EBV in EBV-positive lymphoma, CAEBV, EBV-HLH and EBV-positive transplant patients, EBV antibody testing results cannot reflect the dynamic changes in EBV load or the activity of EBV infection. The joint detection of EBV DNA in both peripheral blood mononuclear cells and plasma/serum may enhance diagnostic sensitivity and provide a more comprehensive assessment of viral burden, thereby supporting improved diagnosis and longitudinal monitoring of EBV-associated diseases (Semenova et al., 2016). The detection of EBV DNA is widely approached through whole blood analysis, which many researchers consider advantageous. According to the Expert Consensus on Laboratory Diagnosis and Clinical Application of EBV Infections, EBV DNA load in whole blood samples is mainly used for the diagnosis of EBV infection in immunodeficient patients. However, its use is generally not recommended for diagnosing infection in immunocompetent patients. For patients with clinical suspicion of CAEBV, a negative EBV DNA result in serum or plasma should not exclude the diagnosis. In such cases, further testing of peripheral blood mononuclear cells (PBMCs) for EBV nucleic acid load is recommended. EBV DNA load in serum and plasma is a valuable marker for evaluating the therapeutic efficacy of EBV-HLH. In addition, dynamic monitoring of EBV nucleic acid load and timely intervention are crucial for the prevention of EBV-related tumors and PTLD (National Collaborative Group on EB Virus Infection in Children and Editorial Board of Chinese Journal of Experimental and Clinical Virology, 2018). Therefore, appropriate sample selection should be tailored according to the patient’s specific disease status.

Numerous studies have shown that (Wang et al., 2016), (Cohen, 2000; Macsween and Crawford, 2003; Vereide et al., 2014), EBV infection is associated with IM, NPC, lymphoma, and other diseases. In this study, the EBV DNA positivity rates of 10 EBV-associated diseases were primarily analyzed. The top three highest positive rates of EBV DNA are IM, NPC, and nasopharyngeal masses. IM is a major clinical syndrome caused by primary EBV infection, with a typical clinical “triad” of fever, pharyngitis, and cervical lymph node enlargement, which may be accompanied by hepatosplenomegaly, and a typical peripheral blood picture characterized by an increase in lymphocytes and atypical lymphocytes (Chinese Medical Association Pediatrics Branch Infection Study Group and National Children’s EB Virus Infection Collaborative Group, 2021). Current research confirms that the clinical manifestations of IM are diverse and nonspecific, which may lead to diagnostic challenges, resulting in frequent misdiagnosis or underdiagnosis and, consequently, delayed or missed therapeutic intervention (Wang et al., 2019).

Among the 152 patients diagnosed with IM, 95 cases (62.5%) were male and 57 cases (37.5%) were female. Regarding age distribution, the majority of cases were adults, with 99 patients (65.2%) aged between 18 and 41 years, while 53 cases (34.8%) were children and adolescents aged 1 to 17 years. Overall, IM in this study predominately affected males and young adults, with 62.5% of cases occurring in males and 65.2% in the 18–41 years age group. Clinically, the most common manifestations included fever, cervical lymphadenopathy, and pharyngitis, which constituted the predominant symptom triad observed in the patient cohort.

Following EBV infection, the host immune system generates a characteristic antibody response. This study conducted serological analysis on 152 patients diagnosed with IM to assess the pattern of EBV-specific antibody production. The production of specific antibodies is known to be associated with the expression of EBV proteins, including EA-IgA, VCA-IgA, VCA-IgM, VCA-IgG and EBNA IgG antibodies. EA-IgA serves as a key marker of EBV activity and proliferation, typically emerging early during the lytic phase of infection (Qin et al., 2023). EA-IgA testing demonstrates high specificity and can assist in determining the occurrence of nasopharyngeal carcinoma. EA-IgA typically appears in serum 3–4 months post-EBV infection and can persist or a prolonged duration. VCA is a structural antigen synthesized in the late stage of EBV lytic replication and is predominantly present during this phase (Mao et al., 2023). VCA-IgA testing is highly sensitive, and the presence of VCA-IgA is a marker of recent infection, reactivation of infection or chronic recurrent persistent infection, and is a sensitive indicator of EBV infection (Hsu et al., 2020). VCA-IgM appears at the time of the onset of clinical symptoms, but lasts only 4–8 weeks. According to the Expert Consensus on Laboratory Diagnosis and Clinical Application of EBV infection (National Collaborative Group on EB Virus Infection in Children and Editorial Board of Chinese Journal of Experimental and Clinical Virology, 2018), among 152 patients with IM, the positivity rates of EA-IgA and VCA-IgA were 32% (21/65) and 31% (20/64), respectively, and the positivity rate of VCA-IgM was 83% (77/93). This observation may be attributed to the patients being in the initial phase of infection at the time of serological testing, wherein the immunological response had not yet progressed to the stage of detectable EA-IgA and VCA-IgA antibody production (National Collaborative Group on EB Virus Infection in Children and Editorial Board of Chinese Journal of Experimental and Clinical Virology, 2018). Meanwhile, the positive rates of EA-IgA, VCA-IgA, and VCA-IgM were elevated in the EBV DNA-positive cohort relative to the EBV DNA-negative group, indicating a potential association with active viral replication.

EBV nucleic acid testing and serological antibody assessment each possess distinct advantages and limitations. Notably, EBV nucleic acid testing offers rapid turnaround, operational simplicity, and a low risk of laboratory contamination. It is valuable in the diagnosis of primary EBV infection in immunodeficient patients, such as transplant recipients; however, its utility is limited by a low negative predictive value in the context of IM. EBV antibody testing offers high specificity and sensitivity, enabling differentiation between primary and past infections, yet may be confounded by the presence of maternally derived antibodies in young children; In immunodeficient patients undergoing blood transfusion or immunoglobulin therapy, the presence of exogenous antibodies can interfere with serological results, diminishing the reliability and interpretive value of serum-specific antibody testing. Clinicians could choose the most appropriate test based on the patient’s clinical signs and symptoms, and in certain cases, a combined approach may provide the most accurate diagnostic assessment.

Given that a positive EBV DNA test may reflect either active or reactivating infection, to better estimate the prevalence of active EBV infection among the 152 patients with clinically diagnosed IM, subjects were stratified into EBV DNA-positive and EBV DNA-negative groups according to their virological testing results. Among 152 patients with IM, the frequencies of fever, sore throat, enlarged cervical lymph nodes, enlarged tonsils, hepatosplenomegaly, elevated ALT, elevated AST, and enlarged spleen in patients with IM were higher in the EBV DNA-positive group compared to the EBV DNA-negative group, a finding consistent with those reported by Shen et al. (2023). Considering the potential for EBV infection to contribute to hepatic dysfunction, an analysis was conducted to explore the correlation between EBV-specific antibody profiles and liver function parameters in the 152 patients diagnosed with IM. In this study, ALT levels were most commonly elevated in the IM patients who tested positive for both EA-IgA and VCA-IgA (Table 2). The presence of VCA-IgA and EA-IgA suggests persistent EBV antigen stimulation, which may contribute to liver function impairment during EBV stimulation. The detection of EBV nuclear antigen (EBNA) suggests an advanced stage of EBV infection. Among the IM patients, 99% tested positive for VCA-IgG, 83% for VCA-IgM, and 23% for EBNA IgG, indicating that the majority of cases in this cohort were consistent with primary EBV infection (28). During primary EBV infection, VCA-IgM typically appears concurrently with VCA-IgG, which generally emerges before EBNA IgG, and VCA-IgG and EBNA IgG signify preexisting EBV infection. The large difference in the positivity rates of VCA-IgG and EBNA IgG in this study may be related to individual differences in serologic responses after EBV infection. It has been reported that the expression pattern of EBV antibodies is complex and varied; for example, 5–10% of healthy individuals fail to develop detectable EBNA IgG antibodies after EBV infection, and the proportion of EBNA IgG negativity is even higher in immunocompromised individuals (Dunmire et al., 2015). However, reliance on EBV antibody testing alone presents certain limitations: VCA-IgM and VCA-IgG are frequently utilized as indicators of EBV primary infection, while high-affinity EBNA IgG generally appears during the late convalescent phase and persists indefinitely, serving as a marker of past infection. In immunocompetent patients, EBV-specific antibody testing remains the gold standard for the diagnosis of IM.

Elevated lactate dehydrogenase (LDH) levels are commonly observed in patients with IM (Zidovec Lepej et al., 2003). Among the IM patients included in this study, the percentage of elevated LDH was 69% (70 cases), with the highest percentage of 1- to 3-fold elevations, at 87%. Approximately 10% of individuals with EBV infection exhibit hepatomegaly, with liver function abnormalities observed in up to two-thirds of cases, and jaundice occurring in 5–15% of patients. The pathophysiological mechanism underlying hepatic involvement in patients with IM remains incompletely understood. A study by Hui Zhang et al. reported that liver function abnormalities associated with EBV infection were not correlated with circulating EBV DNA levels, suggesting that the virus may not exert a direct cytopathic effect on hepatocytes (Zhang et al., 2013). Huanrong Hou demonstrated that patients with IM exhibit an imbalance in NK cell subpopulations, characterized by a significant expansion of classical cytotoxic NK cells, a phenomenon potentially mediated by IL-15. This immunological dysregulation may represent one of the key mechanisms contributing to liver injury in IM (Hou and Kang, 2023). In addition, it has been proposed that the marked proliferation of CD8 + T cells plays a significant role in the pathogenesis of hepatic injury associated with EBV infection (Yin et al., 2019).

Due to the rich expression of CD21 molecules on the B cell surface, EBV can mediate entry into B cells by binding the EBV glycoprotein gp350/220 to CD21, thereby establishing B lymphocytes as the primary target for EBV infection in the majority of cases (Arredouani et al., 2014). Human B cells express receptors for EBV. Following entry into the host, EBV primarily infects B lymphocytes, which subsequently triggers the activation and proliferation of Th cells and CD8 + T cells. This immune response leads to a marked increase in both the number and proportion of CD8 + T cells, enhancing the cytotoxic response necessary for eliminating virus-infected target cells (Kawada et al., 2019). CD4 + is a surface marker of helper T cells and plays a crucial role in supporting both cellular and humoral immunity. Therefore, during the immune response to EBV infection, a decrease in the proportion of CD4 + T cells and B cells is commonly observed, accompanied by a significant reduction in the CD4+/CD8 + ratio, consistent with the results of this study. Yang et al. (2023) found that CD3 + CD8 + T-cell counts and CD3 + CD8 + T-cell ratios were significantly correlated with viral load in children with EBV infection. This is consistent with the findings of Shen et al. (2023). In this study, a reduction in the percentage of CD19% of B lymphocytes was observed in both EBV DNA-negative and EBV DNA-positive groups. This decrease may be attributed to the activity of EBV-specific CD8 + T cells, which recognize latent viral antigens expressed on infected B cells and mediate their cytolytic elimination, thereby leading to a decline in circulating CD19 + B cell levels. In this study, we also found that CD8 + CD38+/CD8 + % was 100% elevated in the EBV DNA-positive group, and CD8 + DR + was elevated in 94%; CD38 and HLA-DR are activation molecules on the surface of T cells, and cellular expression of HLA-DR is a sign of cellular activation. It has been pointed out that the expression levels of CD38 and HLA-DR on the surface of peripheral blood CD8 + T cells are positively correlated with serum ALT and AST level. Furthermore, the evaluation of CD38 + and HLA-DR + CD8 + T lymphocyte populations offers a indirect gage of *in vivo* viral burden and, to a notable degree, provides a refined reflection of hepatic injury severity during the acute phase of IM (Liu et al., 2023). In EBV-infected patients, a Th1/Th2 cytokine imbalance is observed (Ji et al., 2023). There exists a paucity of research concerning the expression of CD38 on T cells and CD8 + T cells in the peripheral blood of patients afflicted with EBV or CMV infection (He et al., 2022; Ji et al., 2023). Acute EBV infection induces significantly higher levels of CD38 expression compared to cytomegalovirus infection. The precise role of CD38 in the life cycles of EBV and CMV, as well as its potential function in safeguarding infected cells against nucleotide depletion and apoptosis, remains to be fully elucidated (Zidovec Lepej et al., 2003). An elevated percentage of CD3 + CD38 + cells is observed, predominated in patients infected with EBV and cytomegalovirus (He et al., 2022). An immunophenotypic analysis of T cells in children with IM, based on HLA-DR expression, has revealed significant activation of both CD4 + and CD8 + T cells. Multivariate analysis showed that the percentage of HLA-DR + CD8 + T cells was an independent prognostic marker for IM and significantly correlated with viral load and disease severity (Wang et al., 2021).

In conclusion, over the past decade, the positive rate of EBV DNA at PUMCH has exhibited significant fluctuations, yet it has not demonstrated a consistent upward trajectory. Notably, the infection rate among adults aged 71 years and older remains markedly higher than that observed in younger cohorts. Furthermore, the prevalence of positive cases is appreciably greater in men compared to women. Accordingly, these populations necessitate vigilant surveillance to facilitate the early detection and management of EBV reactivation or associated pathologies. This study demonstrates substantial representativeness, derived from its extensive sample size (exceeding 70,000 individuals), wide age spectrum (ranging from 2 days to 104 years), prolonged duration spanning a decade, and comprehensive inclusion of consecutive cases suspected of EBV infection. Meanwhile, early detection and treatment of EBV-related diseases remain crucial. In clinical diagnosis and treatment, the tailored selection of specimen types holds profound significance. To reduce EBV infection and EBV-related cancer, it is imperative to ascertain whether the cancer originates from an infectious agent, such as EBV (Lieberman, 2014). In future studies, our objective is to broaden the scope of sample data collection and to investigate additional potential risk factors. Moreover, further investigations are warranted to elucidate the interplay among CD8 + T cells, HLA-DR, CD38, and EBV antibodies in patients with IM.

## Data Availability

The raw data supporting the conclusions of this article will be made available by the authors, without undue reservation.
